# Enantioselective Synthesis of Atropisomers by Oxidative Aromatization with Central-to-Axial Conversion of Chirality

**DOI:** 10.3390/molecules28073142

**Published:** 2023-03-31

**Authors:** Clément Lemaitre, Stefania Perulli, Ophélie Quinonero, Cyril Bressy, Jean Rodriguez, Thierry Constantieux, Olga García Mancheño, Xavier Bugaut

**Affiliations:** 1 Aix Marseille Univ, CNRS, Centrale Marseille, iSm2, 13397 Marseille, France; c.lemaitre13@gmail.com (C.L.); ophelie.quinonero@gmail.com (O.Q.); cyril.bressy@univ-amu.fr (C.B.); jean.rodriguez@univ-amu.fr (J.R.); thierry.constantieux@univ-amu.fr (T.C.); 2 Organic Chemistry Institute, University of Münster, Corrensstraße 36/40, 48149 Münster, Germany; perulli@uni-muenster.de; 3 Université de Strasbourg, Université de Haute-Alsace, CNRS, LIMA, UMR 7042, 67000 Strasbourg, France

**Keywords:** aromatic compounds, atropisomers, conversion of chirality, enantioselectivity, oxidation

## Abstract

Atropisomers are fascinating objects of study by themselves for chemists but also find applications in various sub-fields of applied chemistry. Obtaining them in enantiopure form is far from being a solved challenge, and the past decades has seen a surge of methodological developments in that direction. Among these strategies, oxidative aromatization with central-to-axial conversion of chirality has gained increasing popularity. It consists of the oxidation of a cyclic non-aromatic precursors into the corresponding aromatic atropisomers. This review proposes a critical analysis of this research field by delineating it and discussing its historical background and its present state of the art to draw potential future development directions.

## 1. Introduction

### 1.1. Interest of Atropisomers and Synthetic Methods to Prepare Them

Described for the first time 100 hundred years ago [1], atropisomers are conformers that gained enough stability because of the hindered rotation around a single bond. They are, thus, considered configurational stereoisomers [2], in which the bond with hindered rotation becomes a stereogenic axis. A half time life higher than 10^3^ s is usually set as the limit to claim their existence, corresponding to a barrier to rotation of 93 kJ·mol^−1^ at 300 K [2,3]. However, to be synthesized, stored, and used at ambient or biological temperature, higher barriers to rotation of ≥105 kJ·mol^−1^ are generally required. The most frequently encountered atropisomers are biaryl compounds bearing *ortho* substituents to prevent the rotation around the biaryl bond. However, non-biaryl based atropisomers also exist [4,5,6].

**Figure 1:**
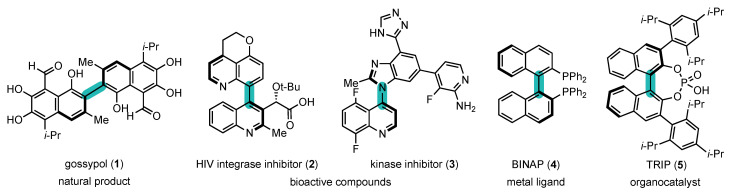
Atropisomers: examples and their fields of application.

In view of their unique properties, atropisomers have received much attention from researchers in all sub-fields of chemistry (Figure 1) [7,8,9]. They are present in natural products [10,11], such as gossypol (**1**) [12], and bioactive compounds [13,14,15,16,17], such as HIV integrase inhibitor (**2**) [18] and kinase inhibitor (**3**) [19], but they also find applications as metal ligands, such as BINAP (**4**) [20], organocatalysts, such as chiral phosphoric acid (**5**) [21], in polymer science [22,23], etc.

Considering the importance of atropisomers and the fact that the two enantiomers are likely to have different behaviors when placed in a chiral environment (as is the case for biological applications), methods to obtain them in enantioenriched form have been actively pursued. While resolution of the racemate is often used, the development of enantioselective synthetic methods can be highly appealing since (i) they obviate the need to discard the unwanted enantiomer or recycle it by enantiomerization; and (ii) they stimulate the creativity of organic chemists.

**Scheme 1:**
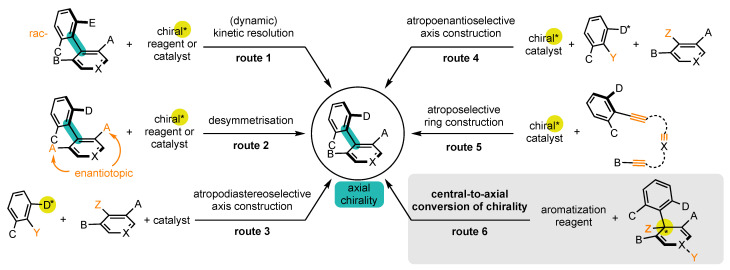
Synthetic strategies to prepare atropisomers (presented on biaryl atropisomers for better readability).

Enantioenriched atropisomers can be synthesized by a variety of methods, which can be classified into six main categories (Scheme 1): [24,25,26,27,28,29,30]

- **Route 1:** Kinetic resolution based on the difference of reactivity of the two atropisomers with a chiral reagent or catalyst. Dynamic kinetic resolution can be envisaged if the two atropisomers are in fast equilibrium in the reaction conditions [31,32,33].
- **Route 2:** Desymmetrization relying on the selective reaction of one enantiotopic group of the prochiral precursor [34,35,36].
- **Route 3:** Atropodiastereoselective construction of the stereogenic axis, for example, by cross-coupling or C-H activation, relying on preexistent stereocenter(s) at the periphery of the aromatic rings [37,38,39,40].
- **Route 4:** Atropoenantioselective construction of the stereogenic axis using a chiral catalyst [41,42,43].
- **Route 5:** Atroposelective ring construction, notably using [2+2+2] cycloaddition or alkyne hydroarylation reactions [44,45].
- **Route 6:** Control of central chirality followed by central-to-axial *conversion of chirality* during aromatization [46,47,48,49].

It should be noted that routes 1 and 2 start from substrates where the bond of the stereogenic axis and the two aromatic rings already preexist. On the contrary, substrates where the bond does not exist are involved in routes 3 and 4, whereas routes 5 and 6 involve substrates where at least one aromatic ring has to be constructed.

Despite the high potential of the central-to-axial conversion of chirality approach [50], no critical survey on the use of this strategy through oxidative aromatization exist in the literature, and we aim to fill this gap with the present review.

### 1.2. Defining Conversion of Chirality

**Scheme 2:**
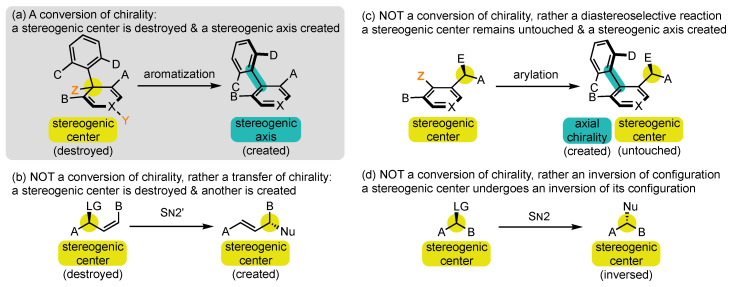
What is and is NOT a conversion of chirality?

We wish to define as *conversions of chirality* [51] all synthetic operations where a stereogenic element is forged in a molecule concomitantly with the destruction of a stereogenic element of a different type (Scheme 2a). The selection of this name requires an in-depth explanation:

1. At first, chirality is used in a quite improper sense, as it is normally applied to a molecule or an object in its entirety, while the process discussed here falls within local stereochemistry discussions [52]. *Conversion of stereogenicity* should, in fact, be recommended, but it seems completely absent from the literature and perhaps more difficult to comprehend for those who are not specialists of this field.
2. *Conversions* of chirality are also referred to in the literature by several other denominations, which all appear to us as less suited. *Transfer* has long been the most frequently used term [53,54,55], but it brings the idea of relocation rather than transformation. For us, it should be restricted to reactions where the stereogenic element that is destroyed and the one that is created are of the same nature, such as in S*_N_*2′ reactions with allylic leaving groups (Scheme 2b) [56]. *Exchange* [49,57] and *interconversion* [47] can also be encountered, but they evoke the idea of reversibility, which is generally not the case in those transformations.

Having defined the conversion of chirality, we now wish to point out which type of phenomenon should NOT be called so. Indeed, all the terms presented above are often misused to describe phenomena that are fundamentally different from conversion of chirality. *Conversions/transfers/interconversions/exchanges of chirality* are by nature potentially stereospecific processes and should be clearly distinguished from diastereoselective reactions. We can, for example, cite those where the configuration of a stereogenic axis is controlled thanks to the presence of one or several stereogenic centers in the surroundings, which remain completely untouched during the transformation (Scheme 2c) [58,59,60,61,62]. Rather, they belong to the strategy exposed in *Route 3* above. Sometimes these terms are even used for reactions that involve only an inversion of configuration, such as in a S*_N_*2 reaction (Scheme 2d) [63].

Being a phenomenon of enantiospecific nature, the efficiency of a conversion of chirality should be evaluated by comparing the relative enantioenrichment of the product and the substrate. In order to normalize this evaluation, Bressy, Bugaut, and Rodriguez introduced the notion of conversion percentage (*cp*) [64], which can be calculated as such and will be used throughout this review:

$$cp=\frac{ee\left(product\right)\times 100}{ee\left(substrate\right)}$$

## 2. Historical Background

### 2.1. A surprisingly Early First Example!

**Scheme 3:**
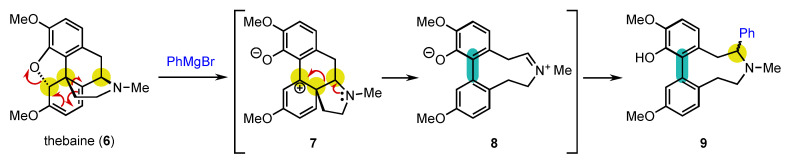
Rearrangement of thebaine: the first example of central-to-axial conversion of chirality during aromatization.

From an historical point of view, it is quite amusing to realize that the first central-to-axial conversion of chirality was achieved in 1905 by Freund, even before the description of axial chirality, during reactivity studies on the natural product thebaine (**6**) (Scheme 3) [65]. Of course, it could not be identified at that moment, and it took almost half a century before the real explanation of what happened could be given by Robinson and Berson [66,67,68]. Treatment of **6** with phenylmagnesium bromide resulted in a complex rearrangement with aromatization to deliver biaryl atropisomer **9** as a mixture of two diastereomers. The reaction proceeds through the opening of the oxa-bridge with rearrangement of the carbon skeleton to afford **7**, which can aromatize to **8**. At this stage, the three initially present stereogenic centers have been destroyed with the concomitant installation of the stereogenic axis. The reaction terminates with trapping of the iminium ion by the Grignard reagent, giving rise to the formation of **9** as two diastereomers, which complicated the understanding of the transformation.

### 2.2. Formulating Research Hypotheses to Achieve Conversion of Chirality

With the understanding of this phenomenon, Berson formulated three fundamental conditions that must be satisfied to allow the synthesis of atropisomers with central-to-axial conversion of chirality [51]:

1. Centrally chiral non-aromatic precursors should be accessible in enantioenriched form;
2. Steric hindrance around the stereogenic axis should be sufficient to ensure its configurational stability;
3. Aromatization should occur with a preservation of the enantiopurity.

**Scheme 4:**
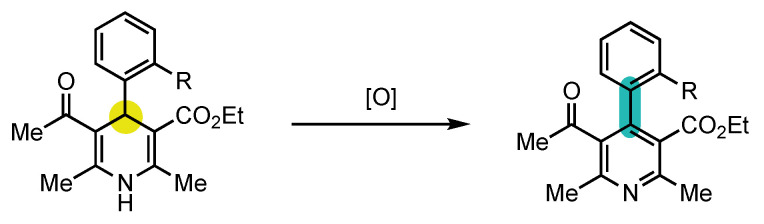
Model proposed by Berson to study aromatization with central-to-axial conversion of chirality.

At that time, Berson could not design a suitable enantioenriched precursor (Scheme 4) and, apart from some other degradation studies in natural product chemistry [69,70,71], the situation remained as such for almost thirty additional years.

### 2.3. Verification of Berson’s Hypotheses by Meyers

**Scheme 5:**
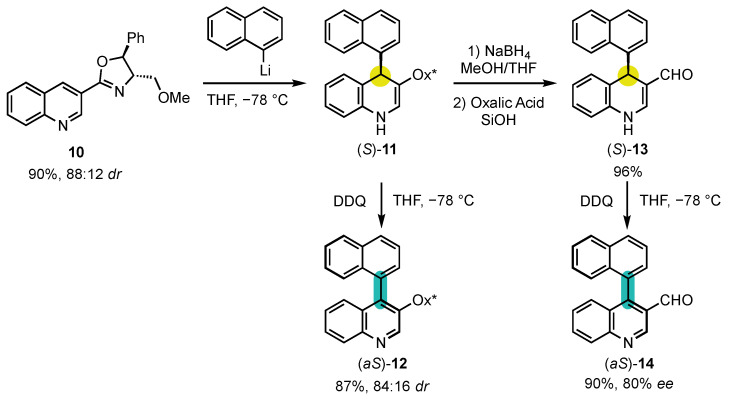
First explicitly designed example of central-to-axial conversion of chirality during aromatization.

Berson’s hypotheses were first confirmed by the explicitly designed central-to-axial conversion of chirality during aromatization proposed by the group of Meyers in 1984 (Scheme 5) [53]. Chiral oxazoline containing quinoline **10** underwent an efficient dearomatization by addition of naphthyllithium to obtain the corresponding dihydropyridine (*S*)-**11** with a diastereomeric ratio of 88:12. The sequence went on with an oxidative rearomatization with DDQ, yielding 4-arylquinoline atropisomer (*aS*)-**12** with a 84:16 *dr*, indicating a conversion percentage of 90%. In order to confirm that the enantioselectivity was not induced by the chiral oxazoline, it was deprotected to afford aldehyde (*S*)-**13**, which was oxidized in the same condition to obtain the pyridine (*aS*)-**14** with an enantiomeric excess of 80%. Those experiments brought the first definitive evidence that central-to-axial conversion of chirality can be an efficient strategy for the preparation of enantioenriched atropisomers.

**Scheme 6:**
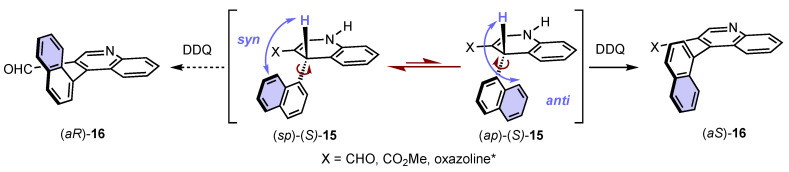
Curtin–Hammett-type description of the stereochemical model during oxidative aromatization with central-to-axial conversion of chirality.

In this seminal work, Meyers also proposed a stereochemical model inspired by Curtin–Hamett principle, which can be applied to all examples that will be discussed in this review [53]. 1,4-Dihydropyridines (DHP) adopt very flat boat conformations [72], with the bulkiest substituent at position 4 on pseudo-axial position to escape gauche interactions with adjacent substituents. On the basis of analyses of the 1,4-dihydroquinoline (*S*)-**15** by X-ray diffraction and NMR, two stable conformers with a rotation barrier below 75 kJ·mol^-1^ could be identified, allowing for a rapid interconversion at room temperature. One conformer is the *anti*-periplanar (*ap*)*-(S*)-**15** in which the non-connective benzene of the napthyl group is below the 1,4-dihydroquinoline core and *anti* to the hydrogen atom, and the other one is the *syn*-periplanar (*sp*)-*(S*)-**15** in which this benzene unit points towards to the hydrogen atom (Scheme 6). Despite the fact that the (*sp*)-*(S*)-**15** conformation is more stable [73], the configuration of the final product (*aS*)-**16** indicated that the (*ap*)-*(S*)-**15** *anti*-periplanar conformation, with the bulky group as far as possible from the hydride to be abstracted, was reacting. This result could be explained by the less important hindrance around the abstracted hydride in this conformation.

### 2.4. Transformations Other Than Oxidations for Conversion of Chirality

Beyond oxidation reactions, such as the one used in the seminal example by Meyers and which will be the focus of this review, other mechanistic pathways are available to achieve conversions of chirality and have been discussed in more general reviews [46,47,48,49,50,74,75,76,77,78,79,80,81,82]. We can notably cite eliminations [83,84,85,86,87,88], retro-cycloadditions [89,90], opening of strained rings [91], and tautomerizations and isomerizations [57,92,93]. To really appreciate the impact of central-to-axial conversion of chirality during aromatization for the synthesis of enantioenriched atropisomers, and the importance of understanding it, one must also realize that in addition to the *explicit* examples presented here, many reactions actually proceed via an *implicit* conversion of chirality [47]. In those transformations, a non-isolable (or non-isolated) reaction intermediate with central chirality undergoes direct aromatization in the reaction mixture to afford an atropisomer. To cite just a few examples, this scenario is present in the enantioselective oxidative coupling of naphtha-2-ols [10,94,95], atroposelective nucleophilic aromatic substitutions [95,96,97,98,99,100], and many recent organocatalyzed transformations [32,101,102,103,104,105,106,107,108,109].

In addition to the previously described examples of oxidative central-to-axial conversion relying on the substrates bearing a chiral auxiliary or derivatives from the chiral pool, recently the attention has been turned towards enantioselective approaches. Among them, the combination of asymmetric catalysis with conversion of chirality has become very popular. In the next sections, we will focus only on enantioselective catalysis–oxidative aromatization with conversion of chirality sequential reactions, and they will be divided considering the type of (hetero)cyclic atropisomer.

## 3. Synthesis of Pyridine Atropisomers

### 3.1. 4-Arylpyridineatropisomers 

**Scheme 7:**
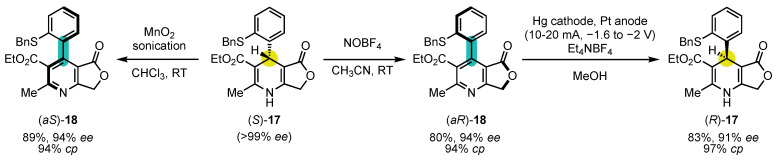
Enantiodivergent oxidation of a 4-aryl-1,4-dihydropyridine in 4-arylpyridine atropisomer and subsequent inversion of configuration of the 1,4-dihydropyridine.

Following Meyer’s initial achievement, other research groups applied similar strategies to the synthesis of pyridine atropisomers. The group of Straub was the first one to expose the strong dependence of conversion of chirality’s efficiency on the choice of the oxidant in 1996 (Scheme 7) [110]. Indeed, starting from enantiopure (*S*)-**17**, obtained by preparative chiral HPLC on chiral stationary phase, they could convert it either to (*aS*)-**18** with 94% *ee* using MnO_2_ or into its antipode (*aR*)-**18,** also with 94% *ee* using NOBF_4_ as the oxidant. This enantiodivergence is likely to arise from the difference in mechanisms and steric requirements between the two oxidants. It should also be noted that other oxidants gave moderately or poorly efficient conversion of chirality (for example: 83% *ee* in favor of the (a*R*) enantiomer for KMnO_4_ or 18% *ee* in favor of the (a*S*) enantiomer for Cu(NO_3_)_2_). Interestingly, electrochemical reduction of (*aR*)-**18** with axial-to-central conversion of chirality could turn it into (*R*)-**17** with 91% *ee*, achieving a formal inversion of configuration of the 1,4-dihydropyridine. 

In this case, the two conformers of the DHP could be observed, and the X-ray structure confirmed that the *syn*-periplanar (*sp)*-(*S*) conformer is the most stable one. The calculation carried out a few years later showed an energy difference of 15 kJ·mol^−1^ between the two conformations [111]. This follow-up study also disclosed that *N*-oxoammonium salt TEMPO^+^BF_4_^-^ is an appealing oxidant for such processes.

**Scheme 8:**
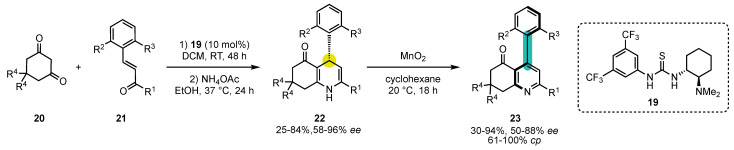
Atroposelective Hantzsch pyridine synthesis by Takemoto’s thiourea catalysis to build chiral 1,4-dihydropyridines/oxidation sequence.

A strong limitation for the implementation of oxidative aromatization with central-to-axial conversion of chirality has long been the preparation of enantioenriched substrates. The key to force this lock was the strategy introduced by Bressy, Bugaut, and Rodriguez in 2016, which combined the organocatalyzed enantioselective synthesis of non-aromatic heterocycles with oxidative aromatization with central-to-axial conversion of chirality [64]. This first example consisted of an enantioselective pyridine Hantzsch synthesis (Scheme 8). Takemoto thiourea catalyst **19** was used to perform the enantioselective Michael addition between cyclic 1,3-diketones **20** and highly congested enones **21**, followed by cyclodehydration in the presence of NH_4_OAc to afford the 4-aryl-1,4-dihydropyridines **22** with good to excellent yields and enantiomeric excesses. Screening of numerous oxidants revealed that only MnO_2_ was efficient at converting the stereochemical information. Cyclohexane was selected as the best solvent to perform the oxidation, and the corresponding 4-arylpyridine atropisomers **23** were obtained with good to excellent *ee* and *cp*. The difficulties in finding an oxidant that delivers high stereospecificities can be ascribed to the fact that **23** possesses one *ortho* substituent on the pyridine ring and two on the other ring, whereas it is the opposite for other systems presented previously.

**Scheme 9:**
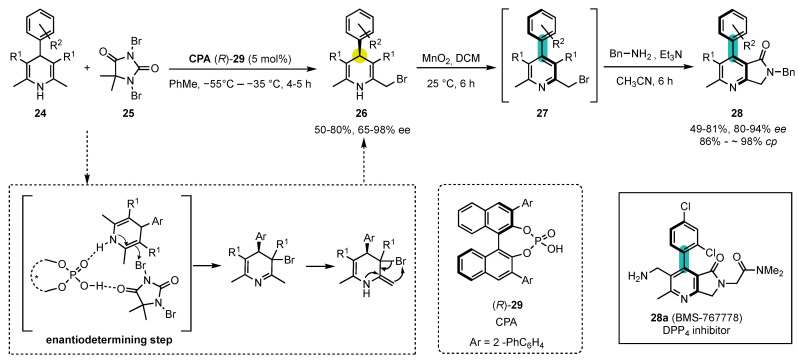
Atroposelective synthesis of 4-arylpyridines from commercially available, symmetric 1,4-dihydropyridines by enantioselective phosphoric acid catalyzed desymmetrization–oxidation–substitution sequence.

More recently, a different approach for the synthesis of axial chiral 4-arylpyridines was developed by Li and Tang from commercially available symmetrical 1,4-dihydropyridines **24** (Scheme 9) [112]. The first step of this synthesis relies on an enantioselective desymmetrization by mono-substitution at one of the methyl groups of the 2,6-dimethyl 1,4-dihydropyridines **24** by C3-bromination—[1,3]-Br atom shift. 1,3-Dibromo-5,5-dimethyl hydantoin (**25**) was used as the brominating reagent, while the presence of the chiral phosphoric acid catalyst **29** allowed for an efficient transfer of chirality to a remote position from the bromination site.

After achieving moderate to excellent yields and enantiomeric excesses (up to 98% ee) in the first bromination reaction, they focused on the following oxidation step to obtain the axial chiral 4-arylpyridines **27** by central-to-axial chirality conversion. As in the previous work, MnO_2_ was selected as the oxidizing agent and DCM as the most suitable solvent for the reaction. 

Finally, the subsequent functionalization of **27** by a SN2/cyclization sequence using different nucleophiles was performed. Thus, when employing amines such as BnNH_2_, the corresponding lactams **28** were obtained in moderate yields (49–81%) and good to high enantioselectivities (80–94% ee) and chiral conversion percentages (86–98% cp). It should also be noted that this strategy opens a route toward the enantioselective synthesis of BMS-767778, a potent DPP4 inhibitor.

### 3.2. Nicotinamide Atropisomers

**Scheme 10:**
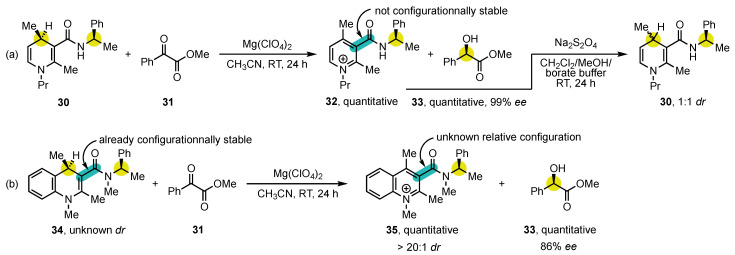
Initial studies on the stereospecific oxidation of NAD(P)H mimics: (**a**) without diastereocontrol of atropisomeric salt **32**, and (**b**) by employment of a configurationally stable substrate.

In addition to 4-arylpyridine atropisomers, 3-carbamoylpyridine congeners, also known as nicotinamides, have also been the center of an intense attention, notably because of their structural similarity to NAD(P)H. Numerous contributions came from the research group of Ohno (Scheme 10). In 1986, they reported the synthesis of the enantioenriched 3-carbamoyl-1,4-dihydropyridine **30** containing a stereogenic center on the amide chain to facilitate analysis by the formation of diastereomers [113]. This substrate was oxidized using methyl phenylglyoxylate (**31**) as a hydride acceptor in presence of Mg(ClO_4_)_2_, delivering highly enantioenriched methyl (*R*)-mandelate (**33**) but with no diastereocontrol of the atropisomeric pyridinium salt **32** (Scheme 10a). This disappointing result was interpreted in terms of low configurational stability, which was confirmed by the fact that the subsequent reduction with Na_2_S_2_O_4_ afforded 3-carbamoyl-1,4-dihydropyridine **30** with 1:1 *dr*. 

To solve this issue, tertiary 3-carbamoyl-1,4-dihydroquinoline **34** was synthesized, and it exhibited a substantial configurational stability at the stereogenic axis, even before oxidation (Scheme 10b). The two diastereomers could be separated by flash chromatography and characterized, even though the relative configurations and the barriers to rotation could not be determined. Treated under the same reaction conditions, it delivered the 3-carbamoylquinolinium salt **35** with >20:1 *dr*. This transformation does not exactly match the definition of the conversion of chirality since the stereogenic axis was already present in the substrate and could, instead, be seen as a stereoablative process [114]. Subsequent studies evaluated the effect of different reaction parameters, such as the nature of the organic oxidant and the metal ion on the reaction outcome, sometimes leading to an inversion of the stereoselectivity [115,116]. Alternative substrates and oxidation methods (Fe(III) or Co(III) anions, electrochemistry) could also be used [117,118,119,120].

**Scheme 11:**
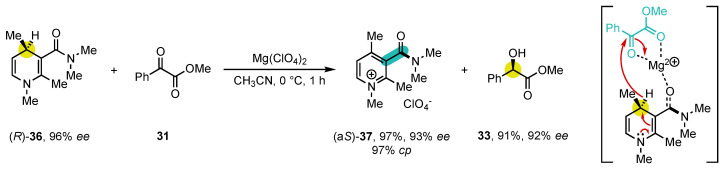
Studies on the enantiospecific oxidation of NAD(P)H mimics.

In 1989, Vekemans and co-workers reported similar studies, but on enantiomeric instead of the diastereomeric systems used by Ohno, simplifying the stereochemical description of the transformation (Scheme 11) [121]. Enantioenriched 3-carbamoyl-1,4-dihydroquinoline (*R*)-**36** was obtained by repeated flash chromatography on chiral stationary phase (cellulose triacetate) until reaching 96% *ee*. Its oxidation by methyl phenylglyoxylate (**31**) delivered configurationally stable pyridinium salt (*aS*)-**37** with an enantiomeric excess of 93%, corresponding to a *cp* of 97%. Its absolute configuration could be determined, which allowed proposing a mechanism where the magnesium salt is complexed by the carbonyl groups of both reaction partners, forcing the C=O of the amide function to point towards the oxidizing reaction partner. Oxidation then proceeds by hydride transfer from position 4 to the *si* face of the activated ketone function. A large diversity of electrophilic compounds were evaluated as hydride acceptors, enabling low (*p*-quinone, acetone), moderate (acetonitrile), or high (benzoin, activation ketones and imines) stereospecificities [121,122,123,124].

## 4. Synthesis of 4-Arylquinoline Atropisomers

**Scheme 12:**
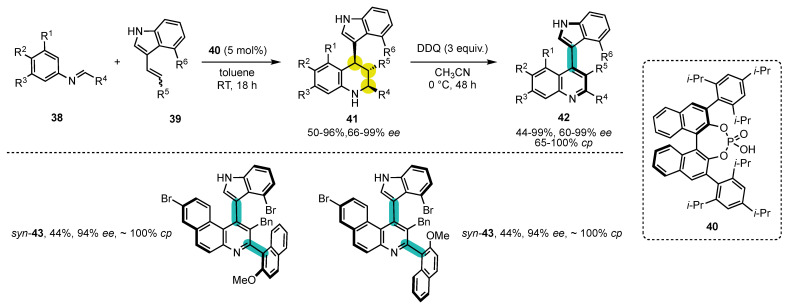
Atroposelective synthesis of indole–quinoline double heterocyclic motives.

On the basis of this seminal report of Bressy, Bugaut, and Rodriguez on the combination of organocatalysis and conversion of chirality [64], the group of Bertuzzi and Corti described the two-step synthesis of chiral indole–quinoline systems (Scheme 12) [125]. The first step consisted of a Povarov reaction between *N*-arylimines **38** and 3-alkenylindoles **39** catalyzed by the chiral phosphoric acid **40** to obtain 1,2,3,4-tetrahydroquinolines **41**, which were then oxidized to the corresponding atropisomeric quinolines **42** using DDQ as oxidant. Good to excellent yields, *ee*, and *cp* were obtained for all examples. In the cases where R^4^ is a bulky aromatic ring, molecules, such as **43,** containing two stereogenic axes, were obtained as separable diastereomers, with good enantioselectivities for both of them.

**Scheme 13:**
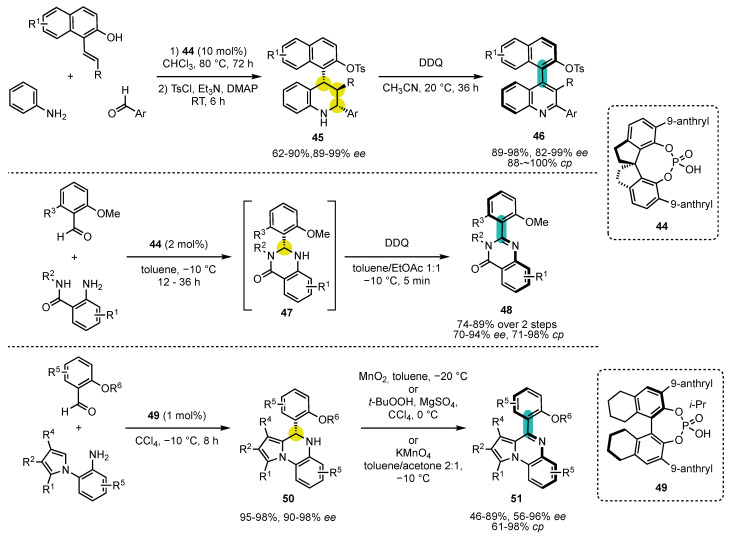
Atroposelective synthesis of 4-naphthylquinolines, quinazolinones, and quinoxalines.

Closely related follow-up studies applied this strategy to the synthesis of other families of nitrogen-containing heterocycles, including 4-naphthylquinolines **46** [126], quinazolinones **48** [127], and quinoxalines **51** (Scheme 13) [128].

All the non-aromatic precursors **45**, **47**, and **50** were obtained by a chiral phosphoric acid-catalyzed annulation or cyclization on in situ formed imines. In the first two cases, the conversion of chirality proceeded smoothly with DDQ as the oxidant with high levels of *cp*. A few examples of the 4-naphthylquinoline series also exhibited two stereogenic axes. However, for the last family of compounds, not only DDQ but also other oxidants, such as *m*-CPBA or PhI(OAc)_2_, were not suitable, leading the authors to develop three sets of reaction conditions which showed strongly different efficiencies depending on the substrates: (i) MnO_2_, toluene, −20 °C; (ii) *t*-BuOOH, MgSO_4_, CCl_4_, 0 °C; and (iii) KMnO_4_, toluene/acetone 2:1, −10 °C. For some examples, changing the oxidation conditions inversed the absolute configuration of the major enantiomer of the final product.

**Scheme 14:**
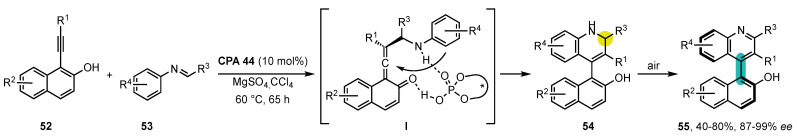
Construction of chiral 4-naphthylquinolines via in situ generated vinylidene–quinone methine intermediates.

In 2022, the group of Zhou designed an elegant alternative approach for the preparation of atropoisomeric 4-naphthylquinolines by chiral phosphoric acid (CPA) catalyzed asymmetric intramolecular cycloaddition of in situ formed vinylidene–quinone methines (VQM) from alkynylnaphthols **52** and isolated or one-pot-generated imines **53** (Scheme 14) [129]. A dual hydrogen-bonding activation by the catalyst **44** was proposed, allowing for a subsequent [2+4]-cycloaddition of VQM **I** to **54** and final auto-oxidation under air to build the desired atropoisomers **55** in moderate to good yields and high enantioselectivities (up to 99% *ee*).

## 5. Synthesis of 4-Arylacridinium Atropisomers

**Scheme 15:**
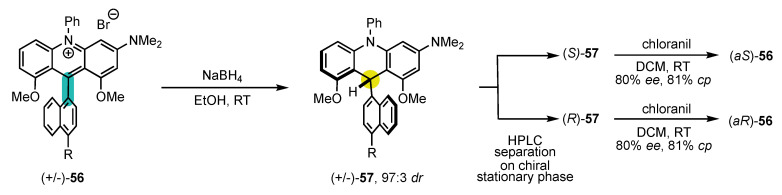
Preparation of enantioenriched 9-arylacridinium salts.

Although the enantioselective synthesis of ionic heterocyclic atropisomers have been much less studied, in 2018, the group of Sparr showed that a similar oxidative approach to the one described for dihydopyridines could be used to prepare cationic atropisomers, namely acridinium salts (Scheme 15) [130]. Racemic 9-arylacridinium salt **56** could be reduced with NaBH_4_ to afford the leuco-form **57**. In this case, both conformers were stable enough to be observed by NMR, and the reduced acridinium was obtained as a racemate with an excellent 97:3 *dr*. Once again, the *syn*-periplanar conformation was the most stable one. The two enantiomers were separated by HPLC on chiral stationary phase and then oxidized with chloranil to yield the enantioenriched 9-arylacridinium salts (a*S*)- and (a*R*)-**56** with an 80% *ee*, corresponding to a *cp* of 81%.

**Scheme 16:**
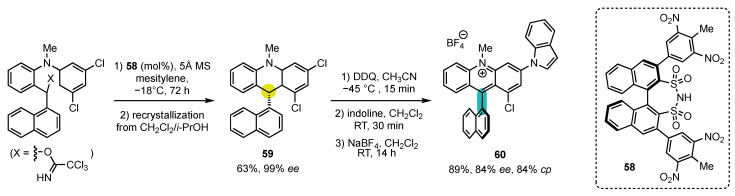
Enantioselective synthesis of atropisomeric 9-arylacridinium salts.

Recently, the same group disclosed an enantioselective synthesis of enantioenriched the 9-arylacridinium **60** combining enantioselective organocatalysis and central-to-axial conversion of chirality (Scheme 16) [131]. The reaction sequence consists of a 6π electrocyclization catalyzed by disulfonimide **58**, followed by in situ oxidation with DDQ. For two examples (one of them presented in Scheme 15), the non-aromatic intermediate **59** was isolated and recrystallized, which allowed evaluating the efficiency of the conversion of chirality. The sequence could even be completed by a regioselective nucleophilic aromatic substitution of one of the chlorine atoms.

## 6. Synthesis of Five-Membered Heterocyclic Atropisomers

Preparing enantioenriched five-membered heterocyclic atropisomers represent a very complicated synthetic challenge because reactivity and selectivity issues are also accompanied by questions on the configurational stability, which is lower than for six-membered congeners.

### 6.1. Oxygen-Containing Heterocycles

**Scheme 17:**
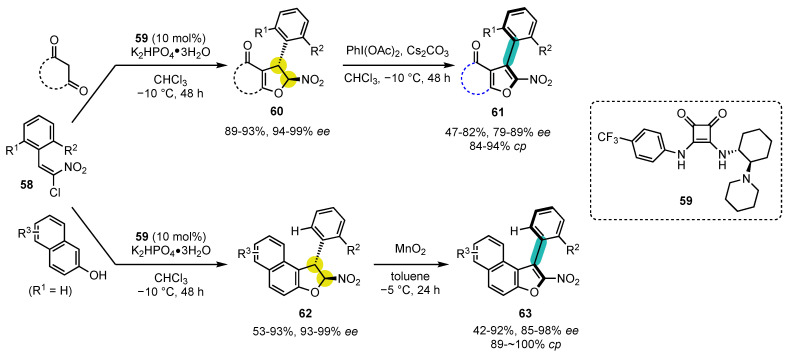
Enantioselective synthesis of atropisomeric 3-arylfurans.

In 2017, Bonne and Rodriguez reported the first enantioselective synthesis of two series of arylfuran atropisomers (Scheme 17) [132]. Using α-chloronitroolefins **61** as Michael acceptors in combination with cyclic diketones or β-naphthols in the presence of squaramide catalyst **62**, two families of dihydrofurans **63** and **65** could be prepared. The reaction consists of an enantioselective Michael addition followed by nucleophilic displacement of the chloride by the oxygen atom. The first family of furan atropisomers **64** was obtained using hypervalent iodine as the oxidant in basic media. For the second family, the best condition for the oxidation of this type of substrate to obtain furan atropisomers **66** was the use of MnO_2_ in toluene.

**Scheme 18:**
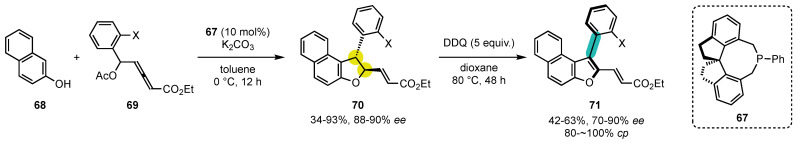
Enantioselective synthesis of atropisomeric 3-arylfurans using a phosphine-catalyzed annulation step.

Similarly, fused furan atropisomers **71** could also be prepared by using a different approach for the organocatalyzed step (Scheme 18) [133]. In this case, allenes **69** react with β-naphthols **68** in the presence of a chiral phosphine catalyst **67** to afford fused dihydrofurans **71**.

**Scheme 19:**
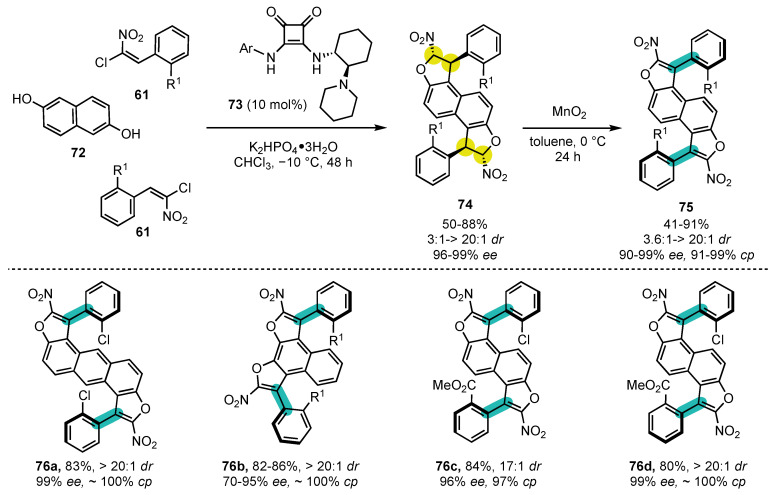
Bidirectional synthesis of bis-benzofuran atropisomers with two distal stereogenic axes.

Subsequently, Bonne and Rodriguez implemented their two-step strategy from α-chloronitroolefins for the bidirectional construction of atropisomeric bis-benzofurans bearing two stereogenics axes (Scheme 19) [134]. Dihydrofurans **74** were obtained in good to very good yields and with good stereoselectivities by enantioselective domino Friedel–Crafts-O-alkylation reaction between 2,6-dihydroxynaphthalene (**72**), and various α-chloronitroolefins **61** catalyzed a bifunctional thiourea **73**. In the case of low diastereoselectivity, the two diastereomers could be separated. The conversion of chirality was then achieved with MnO_2_ in toluene, and the corresponding bis-benzofurans **75** were obtained with good yields and excellent *cp*, higher than 91%. Interestingly, the double axis could be synthesized stepwise to obtain the non-symmetrically functionalized S-shaped oligoarenes **76**. For the second organocatalyzed step, both enantiomers of the catalyst were used to obtain both diastereomers (a*S,*a*S*) and (a*S,*a*R*).

### 6.2. Nitrogen-Containing Heterocycles

**Scheme 20:**
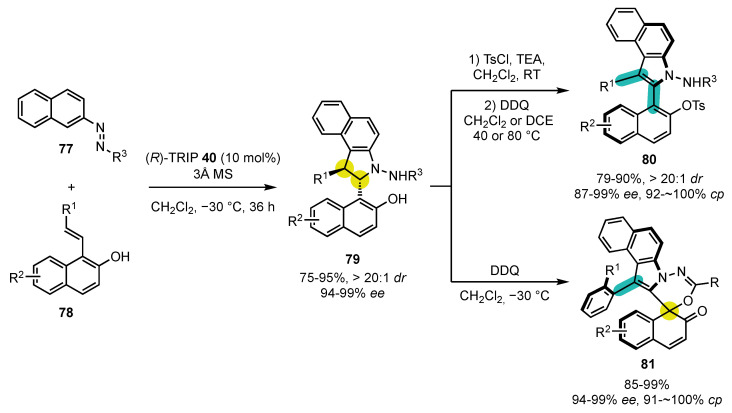
Enantioselective synthesis of atropisomeric 2-arylbenzoindoles.

In 2019, the group of Zhou described the synthesis of 2,3-diarylbenzoindoles containing one or two stereogenic axis (Scheme 20) [135]. The authors used Brønsted acid organocatalysis, using (*R*)-TRIP (**40**) to promote the [3+2] annulation between azonapththalenes **77** and 1-styrylnaphthtols **78** to obtain dihydropyrroles **79** in good yield and excellent diastereo- and enantioselectivities. To avoid possible dearomatization side reactions that would lead to erosion of the stereoselectivity, a sulfonylation of the naphthol moiety was conducted prior to oxidation to the corresponding benzoindole. Initially, a trilfate substitution showed a poor enantioselectivity. This was then notably improved by tosylation, leading to the benzoindoles **80** with high enantioselectivities and with *cp* between 92% and 100% upon oxidation using DDQ in DCE at 40 °C. In the case of R^1^ being an *ortho*-substituted aryl ring, the oxidation proceeded at 80 °C, and molecules with two stereogenic axes were obtained with the same range of *cp* and high diastereoselectivities. DFT calculations carried out on the substrate highlighted the importance of the steric hindrance around the hydride for both conformers to explain the efficiency of the conversion of chirality. Subsequently, the same group utilized directly the free naphthols to undergo a simultaneous central-to-spiro transfer and central-to-axial conversion by exploiting intramolecular hydrogen-bonding interactions between the hydroxy and hydrazine groups to achieve **81** within high levels of enantiocontrol (up to 99% *ee* and 100 *cp*) [136]. 

**Scheme 21:**
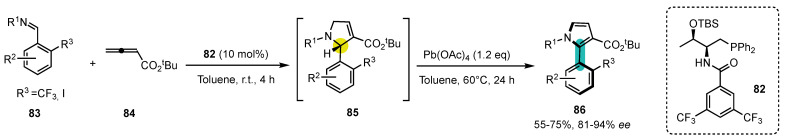
Synthesis of enantioenriched 2-arylpyrrol-based atropisomers.

In 2022, Ullah and Lu reported an approach for the synthesis of CF_3_-substituted 2-arylpyrroles by sequential asymmetric catalytic [3+2] annulation and oxidative central-to-axial chirality transfer (Scheme 21) [137]. To this purpose, a chiral bifunctional amide-phosphine organocatalyst **82** was employed, which allowed the construction of the five-membered N-heterocyclic moiety by reaction between an imine **83** and the allene **84**. Finally, the pyrrole group was built upon oxidation of **85** with Pb(OAc)_4_, which proved superior in terms of chirality transfer to other more commonly used oxidants in chiral-to-axial conversion processes. Following this method, both 2-CF_3_ and 2-iodo-arylpyrroles **86** were prepared in up to 94% *ee*. 

## 7. Synthesis of Carbocyclic Atropisomers

### 7.1. Seminal Results

**Scheme 22:**
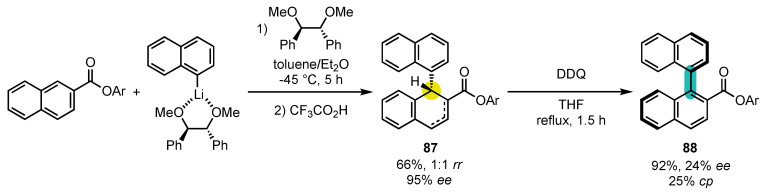
First example of applying oxidative aromatization with central-to-axial conversion of chirality to carbocycles.

As presented in the previous sections, the major part of the literature on oxidative aromatization with conversion of chirality focuses on heterocyclic systems. The first recognized historical example in the carbocyclic series reported by the group of Tomioka in 2009 was disappointing, with a conversion percentage of 25% when oxidizing dihydronaphthalene **87** (existing as two regioisomers) into naphthalene **88** (Scheme 22) [138]. In contrast, the corresponding S*_N_*Ar process, where the aromatization occurs by elimination and not oxidation, was far more efficient [98].

**Scheme 23:**
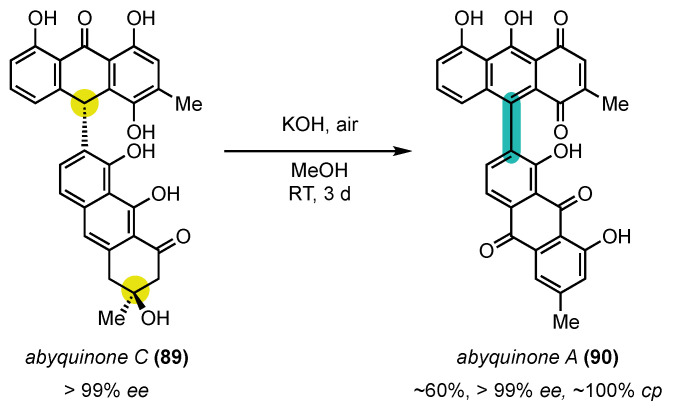
Studies on the aerobic oxidative conversion in the abyquinone family, with central-to-axial conversion of chirality.

On the contrary, efficient conversion of chirality was previously observed by the group of Bringmann when studying a family of natural products called abyquinones (Scheme 23) [139]. Both family members abyquinone C **89** and abyquinone A **90** could be isolated in enantiopure form, the first one bearing central chirality and the second one axial chirality. Their structural resemblance leads to questions about the possibility to oxidize **89** into **90** with central-to-axial conversion of chirality. Aerobic oxidation was indeed observed in basic methanolic solution, with perfect *cp*. Semi-empirical calculations reveal that hydrogen bonding between the phenolic group could lead to conformational blockage on the precursor, explaining the stereospecificity.

### 7.2. Combining Enantioselective Organocatalysis with Conversion of Chirality

It is the use of organocatalysis to prepare enantioenriched non-aromatic precursors that allowed the first general efficient strategies for the synthesis of carbocycles to be designed, even though examples remain scarce compared with heterocycles.

**Scheme 24:**
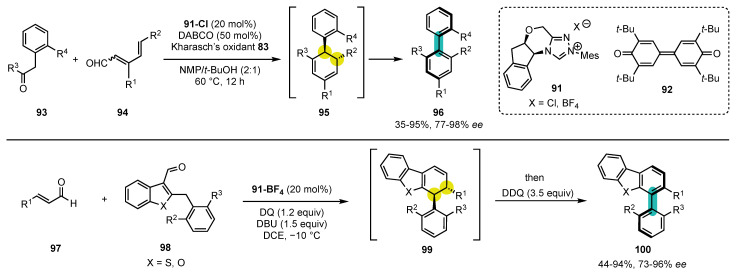
Implicit central-to-axial conversion of chirality during NHC-catalyzed benzannulations by Zhu (**top**) and Zhang and Ye (**bottom**).

In 2019, the group of Zhu made use of enantioselective oxidative NHC organocatalysis with catalyst **91** to prepare chiral cyclohexadienes **95** from carbonyls **93** and α,β-unsaturated aldehydes **94**. These intermediates **95** were readily oxidized into the corresponding atropisomers **96**, with implicit conversion of chirality (Scheme 24, top) [140]. The fact that the intermediate could not be isolated precluded the precise estimation of conversion of chirality efficiency. Interestingly, the stoichiometric Kharasch’s oxidant **92** could be replaced by electrochemical oxidation.

More recently, Zhang and Ye employed a similar approach for the synthesis of axial chiral benzothiophene and benzofuran-fused biaryls **100** (Scheme 24, bottom) [141]. They employed the chiral NHC catalyst precursor **91** as BF_4_ salt for the cyclization between an enal **97** and a 2-benzyl benzoheteroaryl 3-carbaldehyde **98**, in the presence of 1,8-diazabicyclo[5.4.0]undec-7-ene (DBU) as the base and diquat (DQ) as the oxidant, followed by treatment of intermediate **99** with DDQ for the final oxidative aromatization step. The chiral atropisomer products **100** were then obtained in moderate to excellent yields and enantioselectivities (up to 96% *ee*).

**Scheme 25:**
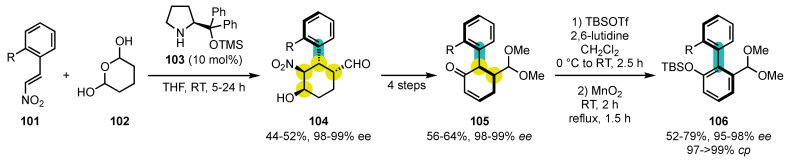
Synthesis of carbocyclic atropisomers by combining aminocatalysis with central-to-axial conversion of chirality.

For the synthesis of carbocyclic atropisomers, the group of Hayashi developed a method relying on enantioselective aminocatalysis (Scheme 25) [142]. It started with a domino reaction of aqueous glutaraldehyde **102** (occurring in its hemiacetal from) with nitroolefins **101**, affording polysubstituted cyclohexane derivatives **104** with four stereogenic centers. These were processed in six steps over intermediate enone **105** to the final biaryl atropisomers **106**. Due to the fact that they could not observe the existence of several conformers for **104**, the authors claimed that the stereogenic axis already exists in the very first chiral product and remains configurationally stable all along the synthesis, hence explaining the very high enantiomeric excess. This scenario appears unlikely, and we believe, instead, that the conformers cannot be observed because they interconvert too rapidly. In this case, the oxidative aromatization would occur with a standard central-to-axial conversion of chirality at the final oxidation step. Further studies on the conformational dynamics of **104** are required to distinguish between the two scenarios. Subsequently, the same group published a related work, where the final aromatization step occurred by tautomerization with, once again, a rather convoluted stereochemical description [143].

**Scheme 26:**
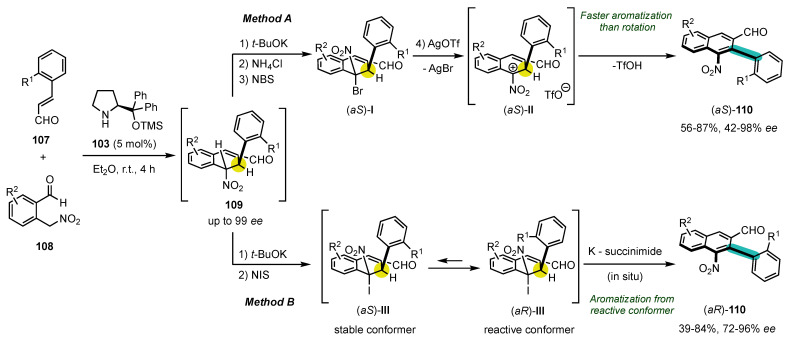
Enantiodivergent multi-step synthesis of atropisomers by Hayashi et al.

In 2021, Hayashi’s group reported an enantiodivergent synthesis of axially chiral biaryls (Scheme 26) [144]. This one-pot synthesis incorporates a multiple-step strategy, starting with an aminocatalyzed one-pot enantioselective Michael reaction and aldol condensation domino reaction between 2-(nitromethyl)benzaldehydes **108** and substituted cinnamaldehydes **107** to give the chiral intermediate **109** in up to 99% *ee*. Later, depending on the halogenating agent and additive used, the synthesis of either one or the other axial conformer could be attained. To achieve the (a*S*)-atropisomer **110**, the intermediate **109** was treated with *t*-BuOK and NH_4_Cl prior halogenation at the C4-position with NBS, followed by reaction with AgOTf. The cationic intermediate **II** is then formed by SN1-type elimination, which is further aromatized by hydrogen atom extraction to build the desired conformers (*aS*)-**110** in good yields (56–87%) and enantiomeric excess up to 98% *ee*.

Alternatively, the (*aR*)-atropisomer **110** could be selectively obtained when reacting **109** with NIS in the presence of the *t*-BuOK, followed by deprotonation with the in situ formed potassium succinimide. DFT calculations showed that, in this case, the conversion of axial chirality in the intermediate **III** is favored toward the reactive species (*aR*)-**III** because the steric hindrance around the acidic H-atom of the initially formed most stable conformer (*aS*)-**III** does not promote the deprotonation. The corresponding atropoisomer (*aR*)-**110** was then achieved in moderate to good yields (39–84%) and enantioselectivities up to 96% *ee*.

**Scheme 27:**
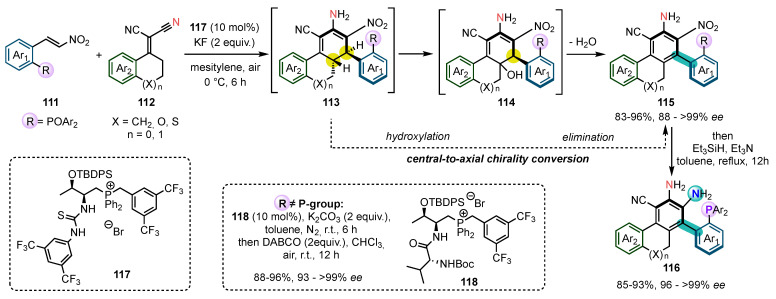
Atroposelective cascade synthesis towards aminophosphine-type ligands.

Recently, in 2022, Wang and co-workers employed an asymmetric thiourea-based phosphonium salt dual catalyst **117** in a cascade procedure for the atroposelective synthesis of aminophosphine-type ligands **116** (Scheme 27) [145]. This represents the first report on the use of such a dual catalyst in a central-to-axial chirality conversion, although the same group already employed it in other catalytic cyclizations [146] as well as for the synthesis of atropisomeric biaryls via kinetic resolution [147]. In this case, the reaction between a nitroolefin **111** and a substituted 2-(3,4-dihydronaphthalen-1(2H)-ylidene)malononitrile **112** provided a chiral intermediate **113** containing four rings. Such an intermediate can undergo a mild hydroxylation with air as the oxidant of choice in the presence of KF to achieve the intermediate **114**, which further undergoes dehydration to build the atropisomer **115**. Thus, the compounds **115** were achieved with remarkable yields (86–99%) and enantioselectivities (88–99% *ee*), which could be then transformed into the N,P-ligands **116** upon concomitant reduction of the nitro and phosphine oxide groups with Et_3_SiH. A similar procedure for other substitution than phosphorus (R ≠ P-group) was also developed, in which the ligand **118** and subsequent use of K_2_CO_3_ and DABCO as bases were required for attaining the same high levels of central-to-axial chirality transfer.

### 7.3. Miscellaneous

**Scheme 28:**
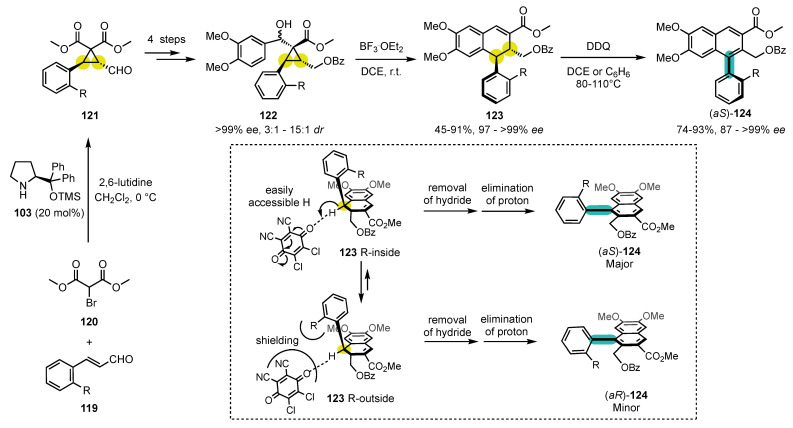
Central-to-axial conversion from chiral cyclopropylcarbinols via dihydronaphthalene intermediates.

In 2022, Nishii and co-workers developed a seven-step procedure to synthetize the (aS)-atropisomers **123** from chiral cyclopropylcarbinols, implying central-to-axial chiral conversion (Scheme 28) [148]. In this first step, the Hayashi-Jørgensen amino catalyst **103** promoted Wang’s asymmetric cyclopropanation between a cinnamaldehyde derivative **119** and dimethyl α-bromomalonate (**120**). The enantiomeric configuration of the formed cyclopropane diester **121** was preserved during the next four synthetic steps to achieve the desired cyclopropylcarbinols **122**. These intermediates undergo a one-pot ring-opening/cyclization in the presence of BF_3_·OEt_2_ to access with a nearly complete steroinduction the chiral dihydronaphthalenes **123**. The last step implies a central-to-axial chirality exchange by oxidative dehydrogenation of **123** using DDQ as the oxidant, providing the atropisomers (aS)-**124** with moderate to excellent enantioselectivities (87–99% ee) depending on the substituent R on the aromatic ring. The thermodynamically most stable conformer with the ortho-substituent pointing outside the naphthyl ring (R-outside) was, again, not the one favoring the last oxidation reaction step due to steric hindrance. Hence, in the intermediate **123** with the R-inside conformation, the hydrogen atom is more accessible and easier to extract, allowing for the oxidative dehydrogenation to take place.

## 8. Control of C-N Atropisomerism

**Scheme 29:**
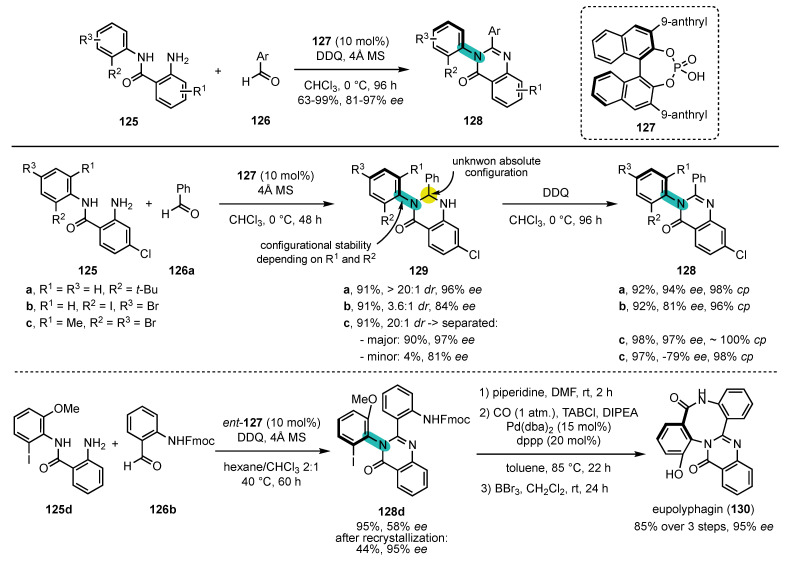
Atroposelective synthesis of quinazolinones with a C-N stereogenic axis.

In addition to the above-presented arylquinazolinones **48** bearing a C-C stereogenic axis (Scheme 13), conversion of chirality was also efficiently applied by the group of Tan for the preparation of enantioenriched related compounds **128** with a C-N stereogenic axis (Scheme 29) [149]. The reaction was carried out in the presence of chiral phosphoric acid catalyst **127** and the oxidant DDQ from the start, precluding the isolation of the non-aromatic intermediate. However, for three examples, the reaction was carried out in a sequential fashion with treatment by DDQ after the isolation of the intermediate **129**. The fact that the absolute configuration of the stereogenic center could not be determined complicates that mechanistic description. However, it appeared clearly that the stereocontrol of the final C-N atropisomerism arose from the initial enantioselectivity obtained during the cyclization step.

Interestingly, three different scenarios could be observed depending on the steric hindrance brought by the *ortho* substituents:

1. With a very bulky *tert*-butyl group in *ortho* position (**128a**), the rotation around the C-N bond is already restricted before oxidative aromatization with very high diastereocontrol, so that this step is not really a conversion of chirality but rather a stereoablative process [114];
2. With a smaller iodine atom (**128b**), the rotation is still possible around the C-N bond before aromatization, resulting in the production of equilibrating diastereomeric conformers, so that the oxidative aromatization fully meets the criteria to be considered a conversion of chirality;
3. When a second *ortho* substituent is present (**128c**), the two diastereomers could be isolated, and each of them was oxidized separately, leading to the two enantiomers of the final atropisomeric product.

Last but not least, compound **128d** could be derivatized in just three steps to afford natural product eupolyphagin **130** in 95% *ee*. It is a very rare example of applying central-to-axial conversion of chirality to the synthesis of atropisomeric natural and/or bioactive products.

## 9. Conclusions and Perspective

Oxidative aromatization with central-to-axial conversion of chirality has a rather special story among strategies that have been elaborated to achieve stereoselective synthesis. Indeed, the first example could be found in the literature even before axial chirality was recognized, even though its understanding was, of course, not possible at that time. Then, its theoretical possibility was hypothesized already in the 1950′s, but it was only three decades later that the experimental realization could be achieved. A decade of rather intense research in this field followed this initial discovery before it slowly went to sleep. Revival arrived approximately 5 years ago with the discovery that enantioselective organocatalysis could act as a very efficient toolbox to prepare cyclic non-aromatic precursors, which could then be aromatized to deliver the corresponding enantioenriched atropisomers. Since then, this strategy was applied to the preparation of diverse families of heterocycles, and less widely to carbocycles.

Our opinion is that oxidative aromatization with central-to-axial conversion of chirality has still not reached the limits of its potential and could be applied to many more atropisomeric structural motives. A current limitation to a broader applicability of central-to-axial conversion of chirality is the little understanding and rationalization of this phenomenon. Indeed, from one publication to the other, a variety of oxidants are used, and even if some of them seem to be privileged (DDQ, MnO_2_), no rather general rule could be drawn. Thus, a trial-and-error approach has to be applied for each new family of precursors. Moreover, there is also a lack of understanding of how the steric and/or electronic properties of the substituents present on the non-aromatic precursor to finely influence the efficiency of the conversion of chirality.

From a preparative point of view, it should also be noted that (super)stoichiometric oxidants have been used in almost every case, and sometimes also highly toxic and/or carcinogenic solvents (for example CCl_4_), limiting the eco-compatibility of this strategy. Future methodologies should target more benign oxidizing conditions, notably catalytic ones. To conclude, research efforts should also be devoted not only to methodological developments but also to its implementation in target-oriented organic synthesis of natural and/or bioactive products.

## Data Availability

Not applicable.
